# Use of and Beliefs About Mobile Phone Apps for Diabetes Self-Management: Surveys of People in a Hospital Diabetes Clinic and Diabetes Health Professionals in New Zealand

**DOI:** 10.2196/mhealth.7263

**Published:** 2017-06-30

**Authors:** Leah Boyle, Rebecca Grainger, Rosemary M Hall, Jeremy D Krebs

**Affiliations:** ^1^ Department of Medicine University of Otago Wellington Wellington New Zealand; ^2^ Centre for Endocrine Diabetes and Obesity Research Capital and Coast Health Wellington Regional Hospital Wellington New Zealand

**Keywords:** mHealth, mobile applications, telemedicine, diabetes mellitus

## Abstract

**Background:**

People with diabetes mellitus (DM) are using mobile phone apps to support self-management. The numerous apps available to assist with diabetes management have a variety of functions. Some functions, like insulin dose calculators, have significant potential for harm.

**Objectives:**

The study aimed to establish (1) whether people with DM in Wellington, New Zealand, use apps for DM self-management and evaluate desirable features of apps and (2) whether health professionals (HPs) in New Zealand treating people with DM recommend apps to patients, the features HPs regard as important, and their confidence with recommending apps.

**Methods:**

A survey of patients seen at a hospital diabetes clinic over 12 months (N=539) assessed current app use and desirable features. A second survey of HPs attending a diabetes conference (n=286) assessed their confidence with app recommendations and perceived usefulness.

**Results:**

Of the 189 responders (35.0% response rate) to the patient survey, 19.6% (37/189) had used a diabetes app. App users were younger and in comparison to other forms of diabetes mellitus, users prominently had type 1 DM. The most favored feature of the app users was a glucose diary (87%, 32/37), and an insulin calculator was the most desirable function for a future app (46%, 17/37). In non-app users, the most desirable feature for a future app was a glucose diary (64.4%, 98/152). Of the 115 responders (40.2% response rate) to the HPs survey, 60.1% (68/113) had recommended a diabetes app. Diaries for blood glucose levels and carbohydrate counting were considered the most useful app features and the features HPs felt most confident to recommend. HPs were least confident in recommending insulin calculation apps.

**Conclusions:**

The use of apps to record blood glucose was the most favored function in apps used by people with diabetes, with interest in insulin dose calculating function. HPs do not feel confident in recommending insulin dose calculators. There is an urgent need for an app assessment process to give confidence in the quality and safety of diabetes management apps to people with diabetes (potential app users) and HPs (potential app prescribers).

## Introduction

Diabetes mellitus (DM) requires tight control of blood glucose to minimize complications and mortality [1,2]. However, many people with DM have suboptimal glycemic control [3,4]. Use of mobile phone apps in diabetes management has been shown to modestly improve glycemic control [5-10]. Despite this promise, health apps remain largely unregulated, and diabetes apps have not always had safety approval [11] or incorporated evidence-based guidelines [12,13].

Blood glucose tracking is the most common feature of diabetes apps [5,14], with other features including record of medications, dietary advice, and tracking, such as carbohydrate content calculation, and weight management support [5,11,12,14-16]. Additionally some apps recommend insulin dosing based on users inputs of glucose levels and estimated meal carbohydrate. Meta-analysis of 22 trials including 1657 patients in which use of mobile phone apps supporting diabetes management was compared to usual care or other Web-based supports showed that app use led to a mean reduction in HbA_1c_ of 6mmol/mol that is 0.5% [9]. This compares favorably with the glucose lowering of lifestyle change, namely diet [17] and oral diabetes medication [18].

However, there are concerns about the appropriateness and safety of apps for diabetes self-management [5,11-13,15]. In 2013 only 1 of 600 diabetes apps reviewed in the USA had received FDA clearance [11]. Similarly a review, specifically of insulin dose calculator apps, determined that only one of 46 calculators was clinically safe. The most common issue was that calculators accepted implausible values for blood glucose readings (eg, negative values), yet would still provide an advised insulin dose [15]. HPs are also concerned about app safety [19] and are advised to take care when advising apps to patients [15]. In the United Kingdom, The Royal College of Physicians Health Informatics Unit (London) has developed a checklist for assessing app quality [19]. However, the multitude of factors HPs must consider while recommending apps, including patient familiarity with technology, app features, ease of use, and FDA approval [19] may be burdensome and not practical in day to day clinical care.

Mobile phone ownership rates are increasing. Similar to trends seen in the United States and Canada, where mobile phone ownership is 72% and 67%, respectively [20], 70% of New Zealanders own a mobile phone, making diabetes apps potentially available to most people [21]. Limited research exists into the use of diabetes apps in New Zealand. However with increasing rates of both diabetes prevalence and mobile phone ownership, access to safe apps is essential for both HPs as potential app prescribers and patients as app users [21,22]. In Scotland, a survey of people with diabetes found high mobile phone ownership (67%) with over half reporting an interest in using apps for self-management of diabetes, but app usage in only 7% of responders [23]. The objectives of this study were (1) To establish whether people with diabetes use apps to assist with diabetes self-management and which features are useful or desirable, and (2) To establish whether HPs treating people with diabetes recommend diabetes apps, which features were thought to be useful, and which features were they confident to recommend.

## Methods

### Study Design and Sample

This cross-sectional observational study used two surveys (see Multimedia Appendices 1 and 2), one for people with diabetes attending a secondary care diabetes outpatient clinic and the second for HPs (who treat people with diabetes) attending a national diabetes conference. Both surveys were multi-choice format, collected, and managed using REDCap electronic data capture tools. REDCap (Research Electronic Data Capture) is a secure, Web-based app designed to support data capture for research studies [24]. The survey questions were derived from criteria in the Mobile app rating scale [25] to address attitudes and practices of both the people with diabetes and HPs. The list of apps was compiled by searching Apple and Android App stores and included the first consecutive ten diabetes apps. We eliminated any apps not specific to diabetes by reviewing app store descriptions. We reviewed the main features from these apps to develop the list of app features. The patient survey asked responders to select any useful app features from a list. Responders could select more than one useful app feature. The HP survey listed app features and used a scale to assess usefulness of app features (from 1 [not at all useful] to 5 [extremely useful]) and their confidence in recommending apps (from 1 [not at all confident] to 5 [extremely confident]).

### Patient Survey

The 1177 people with diabetes attending clinics at Capital and Coast District Health Board (CCDHB), Wellington, New Zealand over a 12-month period (10th September 2014 to 10th September 2015) were the sample population. Out of the total patients, 521 patients with an email address in the hospital management system were invited to participate via email. To include a representation of people without a recorded email address in the sample (n=656), every 5th person was telephoned (up to twice) and invited to provide an email address. Of the 131 patients telephoned, 54 (41.2%) were reached, of whom 49 (91%) agreed to participate. Patients without phone numbers or unable to provide an email address were excluded. This generated a sample population of 570 people.

The survey was piloted with the first 30 patients with an email addresses (chronological order of clinic visits). Responses were reviewed after response rate reached 50%. As 4 questions were unanswered by some participants, a “none of the above” option was added. The invitations were sent out to the remaining 540 participants. A further 31 participants were excluded (4 email address errors, 13 gestational diabetes, 10 deceased, 4 did not have diabetes) resulting in a final total of 539 participants. This survey remained open for 3 weeks, with reminders sent to non-responders at one week and two weeks.

### Clinical Variables

Additional data on all patients were collected from the hospital management system, including age, and the most recent values within the previous 12 months from date of survey for blood pressure (BP), glycated hemoglobin (HbA_1c_), urinary microalbumin to Creatinine ratio (ACR), low density lipoprotein cholesterol (LDL), and total cholesterol to HDL ratio (C:HDL). Prescription of lipid lowering drugs, anti-hypertensive drugs, insulin, or other hypoglycemic medication were also extracted from the medication list from the last visit within the sample period. Type of diabetes was self-reported in the survey (type 1 [T1DM], type 2 [T2DM], other or unknown) and in four participants who had selected ‘other’ or ‘unknown’ diabetes type was determined by examination of the clinical records. For categorization of participants by app use, 4 responders who did not indicate if they had a mobile phone or not were included in the non-app group.

### Health Professionals’ Survey

To obtain data on HPs’ knowledge and recommendation of apps to people with diabetes, a second survey was conducted of the HPs attending the annual scientific meeting of the New Zealand Society for the Study of Diabetes (NZSSD) in May 2016. Immediately prior to the meeting all registered attendees (n=286) were invited to participate in the online survey via email. The data from the patient survey was presented at the conference in a 15-min oral presentation and attendees were encouraged to complete the survey. Paper copies of the survey were also available at the meeting. This survey remained open for 2 weeks, with a reminder sent at 1 week.

### Data Analysis

Data were imported into SPSS version 24 (IBM). Incomplete responses were included in the analysis. In the patient survey, independent sample *t* tests were conducted to compare mean clinical variables (age, BP, C:HDL, LDL, HbA_1c_) by type of diabetes, method of recruitment, and whether the responder used a diabetes mobile phone app. Adjustment was made for unequal variances. Normal distribution was assumed for all variables, apart from urinary microalbumin to creatinine for which a Wilcoxin test was used. No statistically significant differences in these variables or in mobile phone app use were found between patients with recorded email addresses and patients phoned for their email address. Therefore, all 189 responses were combined for further analysis. Chi-square tests were used to compare medications and survey responses by type of diabetes. Statistical significance was determined by exact 2-sided *P* values less than .05. In the HP survey, mean values on the usefulness and confidence Likert scales were calculated to compare app features.

## Results

### Patient Survey

#### Demographics

The survey was completed by 189 of the 539 patients (35.0% response rate, 158/491 from participants with email addresses, 31/48 from telephone contact). Table 1 shows the characteristics of responders. Responders (N=189) were older, with a mean age of 50.0 years (SD 15.7) than non-responders (N=350), who had a mean age of 45.9 years (SD 16.1; *P*=.004) and had lower HbA_1c_ of 62.2 mmol/mol (SD 14.0) (7.8, SD 1.1%) than non-responders (N=325) with mean of 68.9 mmol/mol (SD 18.2; 8.5, SD 2.3%; *P*<.001). There were no significant differences in the rate and type of anti-hypertensive, lipid lowering, and anti-hyperglycemic medications used between responders and non-responders (*P*=.28, −.32, and −.17, respectively). Clinical variables by type of diabetes are shown in Table 2. As expected, responders with T1DM were more likely to be on Insulin than those with T2DM (*P*<.001) whereas responders with T2DM were more likely to be on anti-hypertensive (*P*<.001) and lipid lowering medication (*P*<.001).

**Table 1:** Characteristics of patients completing the survey (n=189).

Characteristic		n (%)
**Type of diabetes (n=189)**		
	T1DM^a^	105 (55.5)
	T2DM^b^	83 (43.9)
	Monogenic	1 (0.5)
**Sex (n=189)**		
	Male	108 (57.1)
	Female	81 (42.8)
**Ethnicity^c^ (n=188)**		
	European^d^	167 (88.8)
	Māori and Pasifika	14 (7.4)
	Indian	8 (4.2)
	Chinese	1 (0.5)
	Other^e^	7 (3.7)
**Education (n=188)**		
	Postgraduate degree	37 (19.6)
	Bachelor’s degree	64 (34.0)
	Apprenticeship	4 (2.1)
	Polytechnic	35 (18.6)
	High school graduate	29 (15.4)
	Some high school	19 (10.1)

^a^T1DM: type 1 diabetes mellitus.

^b^T2DM: type 2 diabetes mellitus.

^c^Responders could identify with >1 ethnicity.

^d^European includes both New Zealand European and other white ethnicities.

^e^Unidentified (n=3), Sri Lankan (n=1), South African (n=1), Tuvaluan (n=1), Native American (n=1).

**Table 2:** Clinical variables among responders by type of diabetes.

Clinical variable		Value^a^ (n)			*P*
		All responders (N=189)^b^	Responders with T1DM^c^ (n=105)	Responders with T2DM^d^ (n=83)	
**Age (SD)**		50.0 (15.7)	43.5 (14.9)	58.4 (12.3)	<.001
	years	189	105	83	
**SBP^e^ (SD)**		127.3 (18.8)	122.8 (14.8)	132.9 (21.7)	.004
	mmHg	124	69	55	
**DBP^f^ (SD)**		75.3 (10.5)	74.0 (9.0)	77.0 (11.9)	.14
	mmHg	124	69	55	
**HbA_1c_^g^ (SD)**		62.2 (14.0)	61.2 (11.4)	63.7 (16.7)	.27
	mmol/mol	7.8 (1.1)	7.7 (1.0)	8.0 (1.4)	
	%	180	101	78	
**LDL^h^ (SD)**		2.3 (0.9)	2.4 (0.8)	2.1 (0.9)	.03
	mmol/L	166	93	72	
**C:HDL^i^ (SD)**		3.1 (1.3)	2.6 (0.9)	3.8 (1.6)	<.001
		168	93	74	
**ACR^j^ (Range)**		0.8 (0.1-527.2)	0.6 (0.1-97.4)	1.9 (0.2-527.2)	<.001
		174	98	75	

^a^Mean (SD) is used for age, SBP, DBP, HbA_1c_, LDL and C:HDL. Median (range) is used for ACR.

^b^All responders includes 1 patient with monogenic diabetes.

^c^T1DM: type 1 diabetes mellitus.

^d^T2DM: type 2 diabetes mellitus.

^e^SBP: systolic blood pressure.

^f^DBP: diastolic blood pressure.

^g^HbA_1c_: glycated hemoglobin.

^h^LDL: low density lipoprotein cholesterol.

^i^C:HDL: total cholesterol to high density lipoprotein cholesterol ratio

^j^ACR: urinary microalbumin creatinine ratio.

#### Diabetes App Use and Desired App Features

96.2% (181/188) of responders reported owning a mobile phone and 84.0% identified this device as a mobile phone (158/188), (Android 52.6% [80/152], iPhone 44.1% [67/152], Windows 3.3% [5/152]). Of the mobile phone owners 23.4% (37/158) reported using a diabetes app. Over half of app users (54%, 20/37) used the app daily, 22% (8/37) used it for a few days per week, and 14% (5/37) used the app less than weekly; 4 responders never used the app.

Of mobile phone owners, those using diabetes apps were more likely to have T1DM (30/96) than T2DM (n=7/61); (*P*=.006). App users were younger with a mean age of 39.0 years (SD 11.1) compared to non-app users having a mean of 52.5 years (SD 15.6), (*P*<.001). There were no other significant differences in clinical variables between app and non-app users.

The majority of responders were not using diabetes apps (80.4%, 152/189), although 60.5% (89/147) reported they would be interested in trying one. Of the 118 people who answered the question, the reasons for not using an app was not knowing they existed (66.9%, 79/118), feeling confident without one (16.9%, 20/118), discontinued use after having used an app previously 16.9% (20/118).

The features most frequently used by current app users were blood glucose diaries (87%, 32/37), followed by carbohydrate/meal diaries (38%, 14/37) with 22% (8/37) reporting insulin dose calculation devices to be useful (Table 3). Table 3 demonstrates the features app users found useful in their current apps. App users reported the most desired feature for future use in an app was an insulin dose calculator (46%, 17/37; Table 4). Table 5 shows that non-app users reported insulin dose calculators to be the third most desired feature (54.6%, n=83/152). Blood glucose diaries were the most desired app feature amongst non-app users (64.4%, 98/152; Table 5). Non app users with T1DM were more likely to desire an insulin dose calculation device, than non-app users with T2DM, *P*=.01).

**Table 3:** Features app users find useful in their current app.

Feature	Total with app (N=37), n (%)	App users T1DM^a^ (n=30), n (%)	App users T2DM^b^ (n=7), n (%)	*P*^c^
Diary of blood glucose levels	32 (87)	25 (83)	7 (100)	.56
Diary of meals and carbohydrate intake	14 (38)	12 (40)	2 (29)	.69
Reminders to check blood glucose	10 (27)	7 (23)	3 (43)	.36
Calculation device for insulin dose	8 (22)	6 (20)	2 (29)	>.99
Blood glucose level guidelines	7 (19)	5 (17)	2 (29)	.60
Personal details and condition information	6 (16)	4 (13)	2 (29)	.57
Calendar of diabetes appointments	7 (19)	5 (17)	2 (29)	.60
Contact details for your diabetes team	4 (11)	2 (7)	2 (19)	.16
Dietary advice	2 (5)	1 (3)	1 (14)	.35

^a^T1DM: type 1 diabetes mellitus.

^b^T2DM: type 2 diabetes mellitus.

^c^A chi-square test was used for calculating *P* values.

**Table 4:** Additional features app users desire in a future app.

Feature	Total app users (N=37), n (%)	App users T1DM^a^ (n=30), n (%)	App users T2DM^b^ (n=7), n (%)	*P*^c^
Calculation device for insulin dose	17 (46)	14 (47)	3 (43)	>.99
Diary of blood glucose levels	13 (35)	12 (40)	1 (14)	.38
Diary of meals and carbohydrate intake	13 (35)	10 (33)	3 (43)	.68
Reminders to check blood glucose	12 (32)	9 (30)	3 (43)	.66
Contact details for your diabetes team	11 (30)	8 (27)	3 (43)	.65
Calendar of diabetes appointments	11 (30)	10 (33)	1 (14)	.41
Blood glucose level guidelines	8 (21)	6 (20)	2 (29)	>.99
Dietary advice	8 (21)	6 (20)	2 (29)	>.99
Personal details and condition information	7 (19)	5 (17)	2 (29)	.60

^a^T1DM: type 1 diabetes mellitus.

^b^T2DM: type 2 diabetes mellitus.

^c^A chi-square test was used for calculating *P* values.

**Table 5:** Desirable app features for a diabetes app amongst non-app users.

Feature	Total non-app users (N=152)^a^, n (%)	Non-app users T1DM^b^ (n=75), n (%)	Non-app users T2DM^c^ (n=76), n (%)	*P*^d^
Diary of blood glucose levels	98 (64.4)	52 (69)	46 (61)	.31
Calendar of diabetes appointments	87 (57.2)^a^	43 (57)	43 (57)	>.99
Calculation device for insulin dose	83 (54.6)	49 (65)	34 (45)	.01
Contact details for your diabetes team	79 (51.9)^a^	42 (56)	36 (47	.33
Diary of meals and carbohydrate intake	73 (48.0)	40 (53)	33 (43)	.26
Reminders to check blood glucose	63 (41.4)	27 (36)	36 (47)	.19
Blood glucose level guidelines	58 (38.2)	29 (38)	29 (38)	>.99
Personal details and condition information	57 (37.5)^a^	32 (43)	24 (32)	.18
Dietary advice	56 (36.8)	26 (35)	30 (40)	.61

^a^Includes 1 additional patient with monogenic diabetes.

^b^T1DM: type 1 diabetes mellitus.

^c^T2DM: type 2 diabetes mellitus.

^d^A chi-square test was used for calculating *P* values.

### Health Professionals’ Survey

#### Demographics and Health Professional App Recommendation

The HPs’ survey was completed by 115 out of 286 HPs (40.2% response rate, 78 online, 37 paper). Table 6 shows the characteristics of responders. Almost all HPs (96.5%, 111/115) owned a mobile phone and of the 113 who answered, 60.2% (68/113) had recommended an app for diabetes management to a patient. Dieticians were most likely to have recommended an app (83%, 10/12), followed by nurses (66%, 42/64), (*P*=.006). There was no relationship between app recommendation and the number of years of treating diabetes (*P*=.48) or the responder’s age (*P*=.49).

**Table 6:** Characteristics of health professionals completing the survey.

General characteristic		N (%)
**Profession (n=115)**		
	Nurse	65 (56.5)
	Doctor	24 (20.9)
	Dietitian	12 (10.4)
	Podiatrists	6 (5.2)
	Other	8 (7.0)
**Years treating diabetes (n=111)**		
	<1	6 (5.4)
	2-5	31 (27.9)
	6-10	26 (23.4)
	> 10	48 (43.2)
**Age in years (n=112)**		
	21-30	9 (8.0)
	31-40	21 (18.8)
	41-50	34 (30.4)
	51-60	42 (37.5)
	60+	6 (5.4)

#### Useful App Features and Confidence Among Health Care Professionals to Recommend Apps

**Figure 1:**
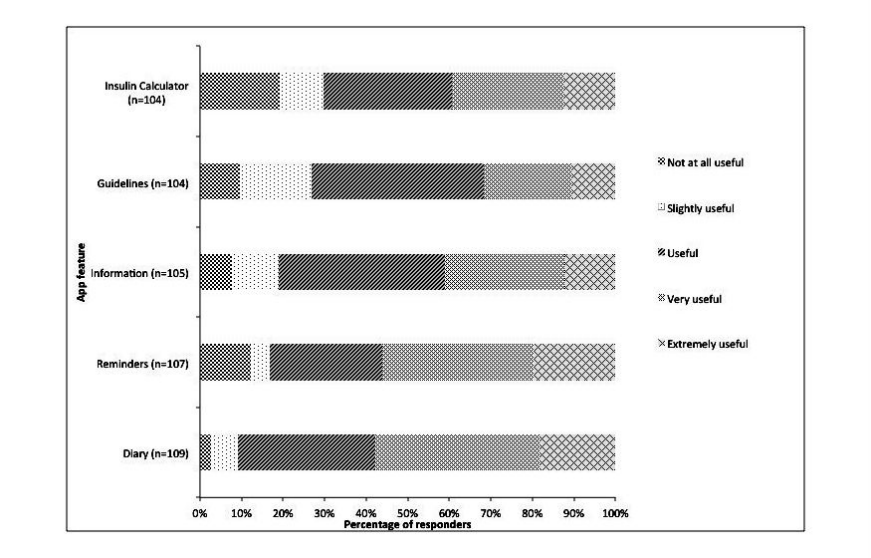
Usefulness of app features reported as useful by Health Professionals.

Overall, all five potential app features were considered useful, with more than 60% of responders selecting that these features were useful, very useful, or extremely useful on the scale of scale 1 (not at all useful) to 5 (extremely useful). Equally, the mean usefulness score was higher than 3 for all 5 features. Blood glucose and carbohydrate intake diaries were rated as being the most useful app feature (Figure 1), with the highest mean score of 3.64 (SD 0.948) for usefulness (Table 7).

**Figure 2:**
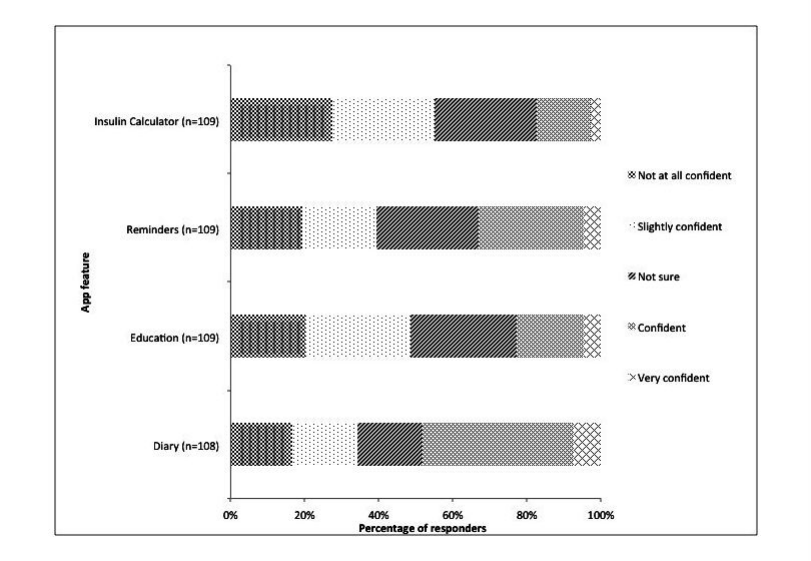
Confidence to recommend app features reported by health professionals.

Glucose diaries were the only type of app which health professionals felt confident to recommend, on an average 3.05 (SD 1.248; Table 7). Responders were the least confident in recommending insulin dose calculators with a mean of 2.38 (SD 1.12) with only 3% of responders being very confident (Table 7 and Figure 2).

**Table 7:** Mean scores for perceived usefulness in app features and confidence to recommend apps by health care professionals.

Usefulness		Confidence	
App type	Mean (SD)	App type	Mean (SD)
Diary^a^	3.64 (.948)	Diary	3.05 (1.248)
Reminders^b^	3.47 (1.216)	Reminders	2.79 (1.187)
Information	3.27 (1.068)	Education	2.59 (1.140)
Guidelines	3.06 (1.068)	Insulin Calculator	2.38 (1.120)
Insulin Calculator	3.03 (1.288)		

^a^Diary includes blood glucose diaries and carbohydrate intake diaries.

^b^Reminders are for medication and checking blood glucose.

## Discussion

### Principal Findings

In this large sample of people with diabetes attending a secondary care clinic in NZ, 19.6% (37/189) of patients reported using diabetes apps to support their self-management. Diabetes app users were younger and more often had T1DM. The most used app feature in current app users was a blood glucose diary (87%, 32/37). The most desirable feature of a future app was an insulin dose calculation function in app users (46%) and a blood glucose diary in non-app users (64.4%). A Scottish survey has reported similar results and observed that people with T1DM were more likely to desire insulin calculators in an app [23].

Almost two-thirds of HPs responding had recommended a diabetes app to patients. Dieticians were more likely to recommend an app than others. Blood glucose and carbohydrate diaries were considered the most useful feature and HPs were most confident to recommend blood glucose diaries. HPs are the least confident recommending insulin dose calculation functions. Over one-third of HPs desire guidance with app recommendations.

### Comparison With Prior Work

Similar to a national American mHealth survey, a large proportion of patients are not using health apps [26]. However, there was a higher rate (20%) of diabetes app use in this patient group compared to the 4% found in a survey of diabetes app use in the USA in 2015 [14] and 7% in Scotland in 2016 [23]. Our findings are consistent with previous surveys showing people using apps are more likely to be younger [26]. It has been suggested that people who are more in need of diabetes care are less likely to use apps [27]; however, we found no significant difference in HbA_1c_ between app users and non-app users. The most favored feature being the blood glucose diary is not surprising given it is the most common feature included in the apps available [5,14]. However some responders are also using health apps that are not specific to diabetes, such as apps for dietary advice.

In contrast with the extensive app problems presented in the literature, over half of the responders with an app reported no problems [5,11-13,15]. This discrepancy may be due to false self-report or responders may have tried multiple apps before finding the one they like. Our study is unable to add significantly to literature about insulin dose calculation problems [15], as only 7 responders reported using their app for insulin calculation. However it is notable that this feature is desired by users and reinforces the importance of having a regulated environment to ensure safety.

The 60.2% of HPs in our survey who had recommended a diabetes app is significantly higher than previously documented amongst physicians across a range of specialties [28], although it is similar to HPs’ recommendation for any type of health app [19]. We did not observe any effect of HPs’ age on app recommendation, although it is previously well established that younger HPs are more likely to adopt mHealth for diabetes [28].

### Strengths and Limitations

A large patient sample size was obtained by contacting all patients seen in the last 12 months with an email address. The risk of overrepresentation by more technology-literate responders through recruitment via email was minimized by also recruiting via telephone and by providing paper surveys at the HPs’ conference. The demographic and clinical data of responders and non-responders were compared, and most variables showed no difference. Responders were actually older than non-responders and had better glycemic control. This study focused on the beliefs and opinions of people with diabetes (potential app users) and HPs (potential app prescribers) rather than simply describing apps for diabetes *.* It is one of the first papers to describe app use in people with diabetes in New Zealand.

This patient sample came from patients in secondary care diabetes clinics, and therefore, app use may be different amongst patients managed in primary care. Similarly, findings may not generalize to patients with poorer glycemic control as responders had statistically significantly lower HbA_1c_ than non-responders. This was a cross-sectional survey that is useful to assess app use at one point in time, but it is likely that people vary their app use and recommendations over time. It was therefore not possible to assess whether the introduction of an app has significant effect on clinical outcomes. Our study did not address the difference in needs in app features between responders on insulin and those not on insulin. Overall the response rates for both surveys were low and responses were limited by self-report and therefore liable to responder bias.

### Conclusions

This study shows app usage is relatively low among people with diabetes, while 60.2% of HPs have recommended an app to patients. There is, however, interest amongst people with diabetes and HPs to use diabetes apps, with strong interest in an insulin dose calculator. Apps with this feature have the potential to improve diabetes control. However, the critical problem of app safety remains a barrier to the prescription and use of insulin dose calculators. Further work is needed to ensure apps are safe and provided in a regulated environment. An app assessment process would provide HPs with confidence in the apps they recommend and would ultimately ensure app quality and safety for app users. At present, however, app users and HPs must remain cautious with diabetes apps, especially those in the insulin dose calculator category.

## Appendix

Multimedia Appendix 1 Survey for people with diabetes mellitus attending diabetes outpatient clinic.

Multimedia Appendix 2 Health professional survey for New Zealand Society for the study of Diabetes.

## Abbreviations

ACR: urinary microalbumin creatinine ratio
Apps: applications
BP: blood pressure
DM: diabetes mellitus
FDA: Food and Drug Administration
HbA _1c_: glycated hemoglobin
C: HDL ratio: total cholesterol: high density lipoprotein cholesterol ratio
HP: health professional
LDL: low density lipoprotein cholesterol
mHealth: mobile health
NZ: New Zealand
T1DM: type 1 diabetes mellitus
T2DM: type 2 diabetes mellitus
