# Supplementary material for: Use of and Beliefs About Mobile Phone Apps for Diabetes Self-Management: Surveys of People in a Hospital Diabetes Clinic and Diabetes Health Professionals in New Zealand
Source: JMIR Mhealth Uhealth. 2017 Jun 30;5(6):e85. doi: 10.2196/mhealth.7263 (PMC5511364; doi:10.2196/mhealth.7263)
Supplement: Multimedia Appendix 1 [file mhealth_v5i6e85_app1.pdf]

Diabetes Self Management and Smartphone Apps

Thank you for taking part in this Questionnaire (approx. 10-15 minutes).

At the end you will have the opportunity to view your answers and to choose whether you would like to receive the overall results once they become available.

What type of Diabetes do you have?

- ◯ Type 1 Diabetes
- ◯ Type 2 Diabetes
- ◯ Other
- ◯ Unsure

(Select one response)

What is the highest degree or level of school you have completed? If currently enrolled, chose highest level completed.

- ◯ No schooling completed
- ◯ Primary/Intermediate School
- ◯ Some High School
- ◯ High School graduate
- ◯ Polytechnic/diploma
- ◯ Apprenticeship
- ◯ Bachelor's degree
- ◯ Postgraduate degree

(Select one response)

Which ethnic group(s) do you belong to?

- ☐ MÄ•ori
- ☐ NZ European/Pakeha
- ☐ Samoan
- ☐ Cook Island Maori
- ☐ Tongan
- ☐ Niuean
- ☐ Chinese
- ☐ Indian
- ☐ Other

(Select as many that apply)

Other, specify

____

(Please specify your ethnic group)

If you have concerns regarding your diabetes management where do you go first for assistance?

- ◯ Practice Nurse
- ◯ General Practitioner
- ◯ Support group/friends/family
- ◯ Internet
- ◯ Smartphone Application
- ◯ Diabetes specialist team
- ◯ Other

(Select one response)

Other, specify

____

(Please specify your first assistance)

Do you regularly have any of the following problems with your Diabetes management?

- ☐ Feeling symptomatic from low blood sugar
- ☐ Feeling symptomatic from high blood sugar
- ☐ Forgetting to measure blood sugar levels
- ☐ Forgetting to take medication or insulin
- ☐ Not known how to identify high or low blood sugars
- ☐ Not knowing whom to contact when in need of assistance
- ☐ Been left without medication/supplies
- ☐ Felt unsure about how to calculate your insulin dose
- ☐ None of the above

(Select as many that apply)

	Not confident at all	Somewhat confident	Neutral	Confident	Very confident
How confident do you feel in your ability to manage your diabetes?	◯	◯	◯	◯	◯

Do you have a mobile phone?

- ◯ Yes
- ◯ No

Is this a smart phone?

- ◯ Yes
- ◯ No

What type of smart phone do you have?

- ◯ Apple (iPhone)
- ◯ Android
- ◯ Windows
- ◯ Other

Please specify the type of smartphone ________________________________

Do you currently have installed or use any Apps for your diabetes management?

- ◯ Yes
- ◯ No

What is the name of the App(s) you have installed? You may select multiple Apps.

- ☐ Glucose Buddy
- ☐ iCookbook Diabetic
- ☐ Diabetes App Lite
- ☐ mySugr Diabetes Diary
- ☐ Easy Diabetes
- ☐ DiabetesConnect
- ☐ Diabetic Friendly Recipes
- ☐ Diabetes App
- ☐ Diabetes FAQ
- ☐ Other

(Select as many that apply)

Other, specify ________________________________

What is the name of the App(s) you have installed? You may select multiple Apps.

- ☐ Diabetes:M
- ☐ Diabetes Logbook by mySugr
- ☐ OnTrack Diabetes
- ☐ Diabetes (King Apps)
- ☐ Diabetes Tracker
- ☐ Diabetes Journal
- ☐ Diabeties Diet Chart
- ☐ Diabetes Cookbook
- ☐ Diabetes - Glucose Diary
- ☐ Recipes for Diabetes
- ☐ Other

(Select as many that apply)

Other, specify ________________________________

Have you encountered any of the following problems with your Diabetes App?

- ☐ Crashing of software
- ☐ Inconsistency in results
- ☐ Problems with units
- ☐ Difficulty understanding advice given
- ☐ Results that do not align with other medical advise you have been given
- ☐ No problems
- ☐ Other
- ☐ None of the above

(Select as many that apply)

Other, specify ______________________________

Do you use your App to calculate your insulin dose?

- ◯ Yes
- ◯ No

Have you had any of the following problems with insulin calculation?

- ☐ An insulin dose is given even though you know you didn't enter all required values
- ☐ Miscalculation of insulin dose
- ☐ Other
- ☐ None of the above

(Select as many that apply)

Other, specify ______________________________

Which of the following features do you find useful in your App?

- ☐ Your contact details and condition information
- ☐ Diary of blood glucose levels
- ☐ Reminders to check blood glucose levels
- ☐ Diary of meals and carbohydrate intake
- ☐ Calculation device to determine insulin dose
- ☐ Guidelines of ideal blood glucose measurements
- ☐ Calender of diabetes related appointments
- ☐ Contact details for your diabetes team, GP
- ☐ Dietary advice
- ☐ Other
- ☐ None of the above

(Select as many that apply)

Other ______________________________

Which other features would you find useful in an app?

- ☐ Your contact details and condition information
- ☐ Diary of blood glucose levels
- ☐ Reminders to check blood glucose levels
- ☐ Diary of meals and carbohydrate intake
- ☐ Calculation device to determine insulin dose
- ☐ Guidelines of ideal blood glucose measurements
- ☐ Calender of diabetes related appointments
- ☐ Contact details for your diabetes team or General Practitioner
- ☐ Dietary advice
- ☐ Other
- ☐ None of the above

(Select as many that apply)

Other, specify ______________________________

How often do you use your App?

- ◯ Every time you eat or take medications/insulin
- ◯ Daily
- ◯ A few days per week
- ◯ Weekly
- ◯ Monthly
- ◯ Only when needing guidance
- ◯ Never

(Select one response)

	Not at all useful	Not very useful	Somewhat useful	Very useful	Extremely useful
How useful do you find your App?	◯	◯	◯	◯	◯

How well does your App function?

◯ Does not function
◯ Some functions work, but slow or has technical problems
◯ App works overall, but slow or has technical problems at times
◯ Mostly functional with minor problems
◯ Perfect with no technical problems
(Select one response)

How easy is it to learn how to use your App?

◯ There are no/limited instructions, confusing
◯ Useable after a lot of time/effort
◯ Useable after some time/effort
◯ Easy to learn to use with given instructions
◯ Able to use immediately, simple
(Select one response)

How easy is it to navigate through your App?

◯ Different sections within the App are disconnected
◯ Easy after a lot of time/effort
◯ Easy after some time/effort
◯ Easy but missing minor links
◯ Perfectly easy
(Select one response)

How do you find the layout/design of your App?

◯ Very poor, some options are impossible to locate
◯ Poor, some options are difficult to locate
◯ Satisfactory, few problems with selecting options
◯ Good, able to locate all options
◯ Excellent, logical and clear layout
(Select one response)

You have completed all of the questions. Would you like to receive the results of this audit once they become available?

◯ Yes
◯ No

What is/are the reason(s) you don't have an App?

☐ Didn't know they existed
☐ They do not work on my mobile phone
☐ Cost
☐ Feel confident without one
☐ Have tried one before and didn't like it
☐ Other
(Select as many as apply)

Other, specify

____

Would you be interested in using a Smartphone Application to assist with your diabetes management?

◯ Yes
◯ No
◯ Unsure
(Select one response)

Which of the following do you find useful in your diabetes management?

☐ Internet
☐ General Practioner or Practice Nurse
☐ Diabetes Team
☐ Support groups/Friends/Family
☐ Blood glucose diary
☐ Pamphlets about Diabetes
☐ Other
(Select as many that apply)

Other, specify

____

If you had an App, which features would you find useful?

- ☐ Your contact details and condition information
- ☐ Diary of blood glucose levels
- ☐ Reminders to check blood glucose levels
- ☐ Diary of meals and carbohydrate intake
- ☐ Calculation device to determine insulin dose
- ☐ Guidelines of ideal blood glucose measurements
- ☐ Calender of diabetes related appointments
- ☐ Contact details for your diabetes team, GP
- ☐ Dietary advice
- ☐ Other

(Select as many as apply)

Other, specify

____

You have now completed all of the questions. Would you like to receive the results of this audit once they become available?

- ◯ Yes
- ◯ No
