# Supplementary material for: Use of and Beliefs About Mobile Phone Apps for Diabetes Self-Management: Surveys of People in a Hospital Diabetes Clinic and Diabetes Health Professionals in New Zealand
Source: JMIR Mhealth Uhealth. 2017 Jun 30;5(6):e85. doi: 10.2196/mhealth.7263 (PMC5511364; doi:10.2196/mhealth.7263)
Supplement: Multimedia Appendix 2 [file mhealth_v5i6e85_app2.pdf]

Diabetes Smartphone Application (App) Survey for NZSSD

We are performing a survey to establish whether health professionals treating people with diabetes in New Zealand recommend smartphone applications (apps) to assist diabetes self-management and which features of diabetes apps health professionals consider important.

1. What is your profession?

☐ Nurse
☐ Dietician
☐ Doctor
☐ Podiatrist
☐ Other, please specify: ____________

2. What is your age?

☐ 21-30
☐ 31-40
☐ 41-50
☐ 51-60
☐ 61+

3. How many years has your professional role focused on treating people with diabetes?

☐ <1 year
☐ 2-5 years
☐ 6-10 years
☐ >10 years

4. Do you own a smartphone?

☐ Yes
☐ No

5. Have you ever recommended a smartphone application (app) for diabetes self-management?

☐ Yes. If yes, which smartphone app(s) did you recommend? ___________________________________
☐ No

6.Please rate how useful you think these diabetes app features are for people with diabetes to enhance their diabetes self-management:

	**Not at all useful**	**Slightly useful**	**Useful**	**Very useful**	**Extremely useful**
Information about diabetes e.g. dietary advice, contact information for diabetes team	☐	☐	☐	☐	☐
Individualised guidelines for ideal blood glucose levels	☐	☐	☐	☐	☐
Diary for blood glucose and carbohydrate intake	☐	☐	☐	☐	☐
Reminders to check blood glucose, take medications	☐	☐	☐	☐	☐
Insulin calculation device	☐	☐	☐	☐	☐

7. How confident do you feel in recommending diabetes apps to patients in the following categories?

	**Not at all confident**	**Not confident**	**Not sure**	**Confident**	**Very confident**
Diabetes education apps	☐	☐	☐	☐	☐
Blood glucose diaries	☐	☐	☐	☐	☐
Apps with reminders for medication or blood glucose changes	☐	☐	☐	☐	☐
Insulin calculation devices	☐	☐	☐	☐	☐

8. Thank you for completing the survey. Please add any comments or feedback:
